# Multi-Characterization of Rejuvenators’ Impact on Aged Asphalt Binder Properties

**DOI:** 10.3390/ma18174060

**Published:** 2025-08-29

**Authors:** Sen Hu, Wentao Bu, Kaimin Niu, Haowu Wang

**Affiliations:** 1 School of Civil Engineering, Chongqing Jiaotong University, Chongqing 400074, China; husen@mails.cqjtu.edu.cn (S.H.); wanghw@mails.cqjtu.edu.cn (H.W.); 2 Wanzhou District Highway Affairs Center of Chongqing Municipality, Chongqing 404160, China; buwent93@163.com; 3 Research Institute of Highway, Ministry of Transport, Beijing 100088, China

**Keywords:** aged asphalt, regenerator, regenerated asphalt, microstructural characteristics, rheological properties

## Abstract

Aging of asphalt is a major cause of pavement distress. While regenerators restore aged asphalt, their mechanisms and efficacy differences remain unclear. This study quantified the repair effects of waste bio-oil (WBO) and mineral oil (MO) rejuvenators on aged asphalt binder using a comprehensive characterization approach. Conventional properties (penetration, softening point, ductility), functional groups (FT-IR), thermal stability (TG), differential scanning calorimetry (DSC), and dynamic shear rheology (DSR) were analyzed. Results reveal distinct mechanisms: WBO acts chemically via polar molecules, selectively reducing oxygen-containing groups and significantly improving ductility, while MO acts physically through light components that dilute viscosity, exhibiting weaker chemical repair. WBO-regenerated asphalt showed a lower thermal-oxidative peak temperature, superior low-temperature ductility, and enhanced high-temperature rheological performance (higher rutting factor, optimized viscoelasticity). These mechanistic differences—chemical restoration (WBO) versus physical replenishment (MO)—determine performance outcomes at the binder level. The findings provide a theoretical basis for regenerator selection in pavement engineering, highlighting WBO’s advantages for functional group restoration and balanced thermal rheological properties, supporting sustainable road development.

## 1. Introduction

During long-term service, asphalt pavements undergo irreversible aging due to the combined effects of ultraviolet radiation, oxidation, thermal cycling, and traffic loading. This degradation process leads to asphaltene aggregation and volatilization of light components, resulting in significantly reduced ductility and increased brittleness of the asphalt binder. These material changes ultimately manifest as various pavement distresses, including low-temperature cracking and high-temperature rutting [1,2,3,4]. To address strategic imperatives such as enhancing the recycling of aged asphalt and reducing carbon emissions, the application of rejuvenators to restore the performance of asphalt has emerged as a core technology in sustainable pavement maintenance [5,6,7]. However, the current mainstream regenerants are mainly WBO-based [8] and MO-based [9]. These two types differ in terms of composition, mechanism of action, and repair mechanism. With the global implementation of green development, developing regenerants that balance performance and sustainability has become a key challenge in the field of road engineering.

Current research predominantly focuses on a singular category of rejuvenators. Chang et al. [10] employed SARA (Saturates, Aromatics, Resins, Asphaltenes) fractionation to analyze the chemical composition of rejuvenated asphalt. Their study demonstrated that MO rejuvenators restore pavement performance by replenishing light components lost during aging and thereby optimizing the colloidal structural stability. Yian et al. [11] characterized mineral oil rejuvenated asphalt (MORA) using conventional physical test, and FT-IR. Results confirmed a physical rejuvenation mechanism dominated by softening and permeation processes, with no detectable changes in functional groups. Muhammad et al. [12] systematically investigated microstructural characteristics and rheological properties of MO-rejuvenated asphalt, with particular emphasis on its long-term evolution through accelerated aging simulations. The effect of varied chemical fractions present in WBO on the aging characteristics of recycled asphalt was examined by Zhao et al. [13] Their findings revealed that reduced volatility of light fractions correlates with enhanced anti-aging performance. Qin Y et al. [14] demonstrated that adding light components from waste bio-oil rejuvenated asphalt (WBORA) to aged asphalt can optimize its flow properties while enhancing its self-healing capabilities. Zhang Xintao et al. [15] noted that variations in the sources, diverse compositions, and unstable properties of WBO lead to inconsistent performance in recycled asphalt. When classified by molecular weight, experimental results revealed that larger molecules contribute to greater stability, while smaller molecules improve flow properties. To address the limitations of single components, we conducted a comparative assessment of WBO and MO rejuvenators. However, for asphalt of identical aging levels, comparative studies on these two types of rejuvenators remain insufficient regarding compositional changes, microstructural characteristics, thermal stability variations, and rheological characteristics. Further investigation is needed to understand the differences in the microscopic and macroscopic properties, as well as the long-term functional performance of the rejuvenated asphalt.

Therefore, this study focuses on investigating the similarities and differences in the rejuvenation performance of these two types of rejuvenators on aged asphalt. Two representative rejuvenators were selected: WBO derived from waste vegetable oil and naphthenic MO. The study centered on aged 70-penetration base asphalt, which was produced following aging via the Rolling Thin Film Oven Test (RTFOT) and subsequent Pressure Aging Vessel (PAV) process. The dosage of each rejuvenator was determined to restore the penetration of the aged asphalt to the level of the original base asphalt. At this optimal dosage, conventional physical tests (penetration, softening point, and ductility) were conducted, and comprehensive characterization was performed using FT-IR, TG, DSC, and DSR. This systematic evaluation provides theoretical guidance for selecting and applying these two rejuvenator types in pavement engineering.

## 2. Materials and Methods

### 2.1. Raw Materials

#### 2.1.1. Virgin Asphalt

The SK70# base asphalt (SK Energy Co., Ltd., Seoul, Republic of Korea) was employed as the virgin binder. Conventional properties were determined following Chinese industry standard JTG E20-2011 [16] (penetration: T0604; softening point: T0606; ductility: T0605), with detailed methodology provided in Section 2.3.1. The experimentally measured properties are comparatively presented in Table 1 alongside the manufacturer’s specifications.

#### 2.1.2. The WBO and MO

**Figure 1:**
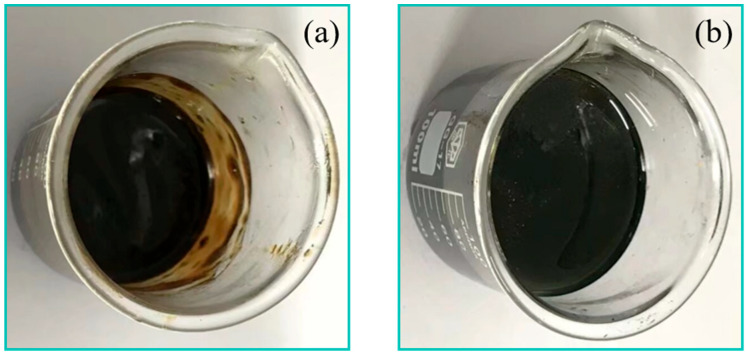
Macroscopic Appearance of Different Rejuvenating Oils: (**a**) WBO; (**b**) MO.

The WBO was sourced from a waste vegetable oil collection facility in Chongqing. It was produced by collecting food residues from local restaurants, followed by filtration and purification to obtain WBO. This waste cooking oil underwent transesterification, after which the glycerol layer was removed via phase separation. Excess methanol was then recovered through distillation, yielding the final low-viscosity WBO. The predominant fatty acid chains are known to be oleic (C18:1), linoleic (C18:2), and palmitic (C16:0) acids, which impart significant polarity and reactivity to the oil due to their ester and carboxylic acid groups. The MO was commercially procured from Xin Tuo Co., Ltd. (Suzhou, China). Regarding the relevant performance indices of both oils, as reported in the literature [17], these indices are specifically presented in Table 2 and Figure 1 to clearly show their performance characteristics and differences.

### 2.2. Preparation of Aged Asphalt and Rejuvenated Asphalt

**Figure 2:**
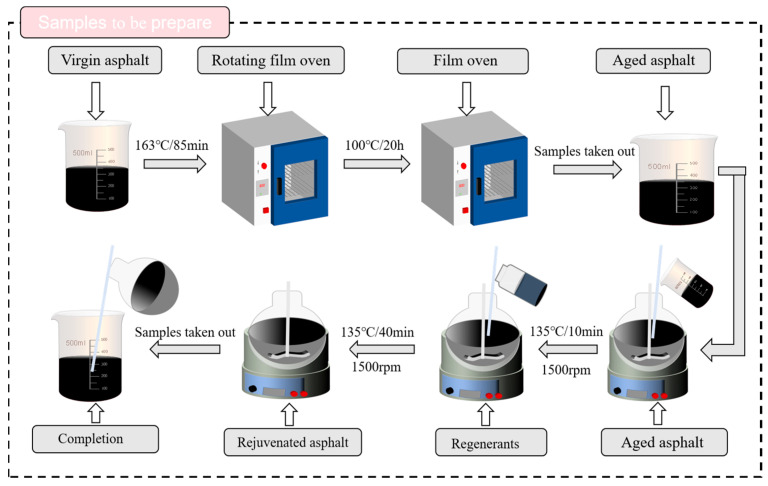
Sample preparation procedure.

The asphalt aging process was systematically conducted using standardized equipment: short-term aging was performed in an RTFOT oven (Model XY-RTFOT-6, Xianyang Instrument, Xianyang, China) at 163 ± 0.5 °C for 85 min with 15 rpm rotation, followed by long-term aging in a PAV system (Model PAV-9200, IPC Global, Melbourne, Australia) at 100 ± 0.3 °C for 20 h under 2.1 ± 0.05 MPa pressure, strictly following ASTM D2872 and D6521 [18] protocols, respectively, with all aged samples subsequently conditioned at 25 ± 1 °C for 24 h prior to testing. The resulting long-term aged asphalt exhibited properties (Table 3) that no longer met specification requirements. Rejuvenated asphalt samples were prepared by blending the aged asphalt with WBO or MO rejuvenators. The mixtures were shear-mixed at 135 °C and 1500 rpm for 40 min. Five rejuvenator dosages (0%, 3%, 6%, 9%, and 12% by weight of asphalt) were evaluated, with corresponding samples designated as 0%, 3%, 6%, 9%, and 12%, as illustrated in Figure 2.

### 2.3. Test Methods for Asphalt Binder

#### 2.3.1. Conventional Performance Tests

Following the test procedures outlined in JTG E20-2011 standards T0606, T0604, and [16], measurements were taken for the conventional properties of asphalt, such as penetration, softening point, and ductility at 10 °C.

#### 2.3.2. Viscosity Test

The viscosity–time relationship was determined using an NDJ-2C rotational viscometer (Shanghai Nirun Intelligent Technology Co., Ltd., Shanghai, China) following ASTM [19] standard test method. A No. 27 rotor and a rotational speed of 80 r/min were employed, with data recorded every 5 s until the viscosity stabilized.

#### 2.3.3. FT-IR (Fourier Transform Infrared Spectroscopy)

A BRUKER TENSOR II Fourier transform infrared spectrometer (Bruker Optics GmbH, Ettlingen, Germany) was utilized to analyze the chemical structure and functional groups of the materials. The sample was prepared into a pellet by mixing with potassium bromide at a ratio of 1:1000. The instrument was set with a resolution of 0.2 c ^−1^, a scanning rate of 16 scans per second, and a scanning range of 4000–400 cm^−1^.

#### 2.3.4. Thermogravimetric Analysis (TG)

A TG analyzer (Model ATS-STA-209, manufactured by NETZSCH, Selb, Germany) was used to evaluate the recycled asphalt. High-purity nitrogen served as the protective gas, with a flow rate maintained at 50 mL/min. The temperature was programmed to range from 28 °C to 550 °C, using a heating rate of 10 °C per minute.

#### 2.3.5. Differential Scanning Calorimetry (DSC)

A differential scanning calorimeter (Model STA 449C/214, NETZSCH, Selb, Germany) was used to test the thermal stability of the recycled asphalt. The test was conducted under a nitrogen atmosphere, with temperatures ranging from 28 °C to 550 °C and a heating rate of 10 °C/min.

#### 2.3.6. Dynamic Shear Rheometer (DSR) Test

**Figure 3:**
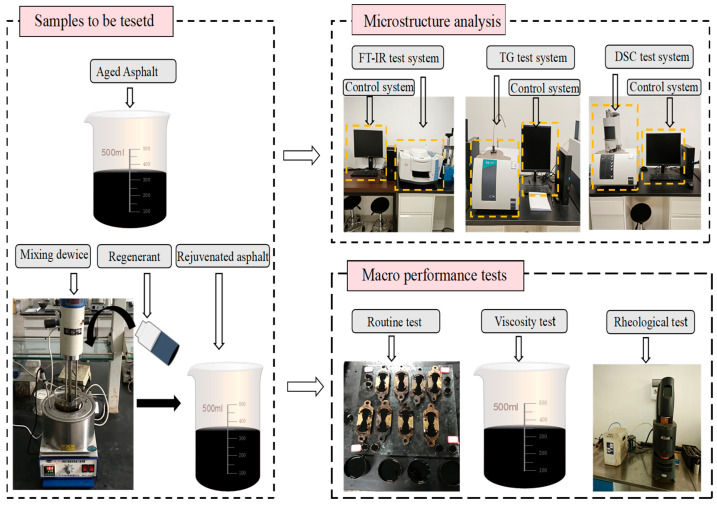
Test methods.

A dynamic shear rheometer (DSR, Model MCR102, Anton Paar GmbH, Graz, Austria) was used to measure the rheological properties of asphalt materials under different temperatures and frequencies. For the temperature sweep test at a fixed frequency, the test temperature ranged from 40 °C to 80 °C, with a scanning frequency of 10 rad/s. A 25 mm diameter rotor was used, with a sample thickness of 1 mm. A strain of 2% was selected for the frequency sweep test, based on the common linear viscoelastic region (LVR) range reported in studies on similar materials [20,21]. For such regenerated asphalt, the LVR is typically observed to be stable within 0.5–5% strain, where the complex modulus (G*) remains independent of strain. Thus, 2% strain was chosen as a conservative value within this empirical range to avoid strain-induced nonlinearity. A frequency sweep test was further performed at the same temperature to acquire rheological parameters such as complex modulus and phase angle. As shown in Figure 3.

## 3. Results

### 3.1. Conventional Properties of the Asphalt

#### 3.1.1. Optimization of Rejuvenator Dosage

**Figure 4:**
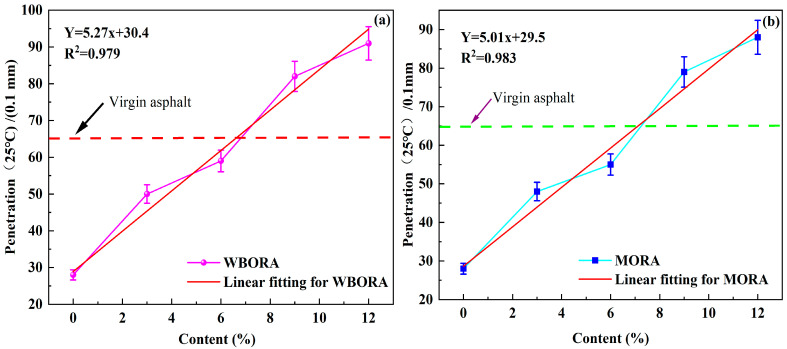
The penetration of aged asphalt with different content of rejuvenators: (**a**) WBORA; (**b**) MORA.

Conventional physical properties characterize asphalt performance [22]. The penetration response of aged asphalt binders to varying rejuvenator mass fractions is quantified in Figure 4.

As shown in Figure 4, compared with the virgin asphalt penetration value of 65 (0.1 mm), the aged asphalt hardened significantly with reduced penetration. The penetration of aged asphalt increased progressively with higher rejuvenator mass fractions. This restoration effect is attributed to the light and medium components [23] in the rejuvenator, which exhibit low molecular weights and high aromatic content. These characteristics enhance the softening efficiency and regeneration capacity of aged asphalt. Excessive or insufficient penetration adversely affects pavement performance. Overly high penetration compromises high-temperature stability, while excessively low values promote pothole formation. Thus, selecting the optimum rejuvenator mass fraction is critical for restoring pavement performance [24]. Using the original asphalt penetration (65/0.1 mm) as the benchmark, linear regression models were established for two recycled asphalts, yielding correlation coefficients of 0.979 and 0.983. The optimum mass fractions calculated from these models were 6.5% for WBO and 7.5% for MO. Comparative evaluation of asphalt regenerated at these optimum dosages was conducted through multi-dimensional assessment of macroscopic properties, microscopic characteristics, and rheological behavior.

#### 3.1.2. Softening Point

**Figure 5:**
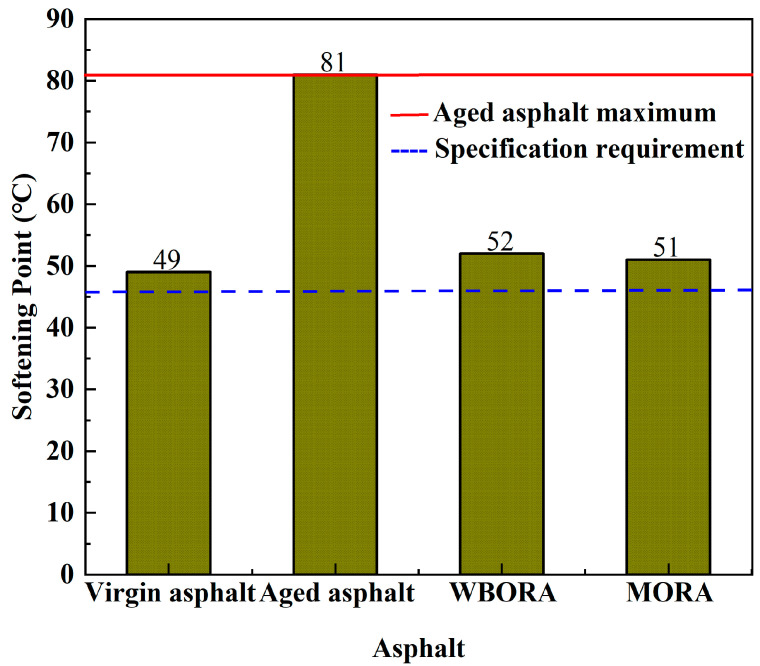
Softening point values of virgin asphalt, aged asphalt, WBORA, and MORA binders. The solid red line indicates the maximum softening point of aged asphalt (81 °C), while the blue dashed line represents the minimum specification requirement (46 °C) according to JTG F40-2004 [16].

Figure 5 reveals divergent softening points in the two recycled asphalts despite identical penetration grades. This discrepancy arises from distinct regeneration mechanisms: (1) For WBORA, polar molecules in WBO partially restore aging damage through hydrogen bonding [25] and π–π stacking [26], forming a moderately cross-linked network that reduces the softening point. (2) For MORA, non-polar hydrocarbons physically dilute asphaltenes, disrupting structural integrity and causing the softening point to approach that of virgin asphalt (ΔT = 30 °C vs. WBORA’s ΔT = 28 °C). The greater reduction in MORA’s softening point indicates superior plasticization efficacy of bio-oil, enhancing high-temperature stability adaptation. Critically, both recycled asphalts exhibit softening points closely matching virgin asphalt, demonstrating precise compensation through tailored rejuvenator design.

#### 3.1.3. Ductility

**Figure 6:**
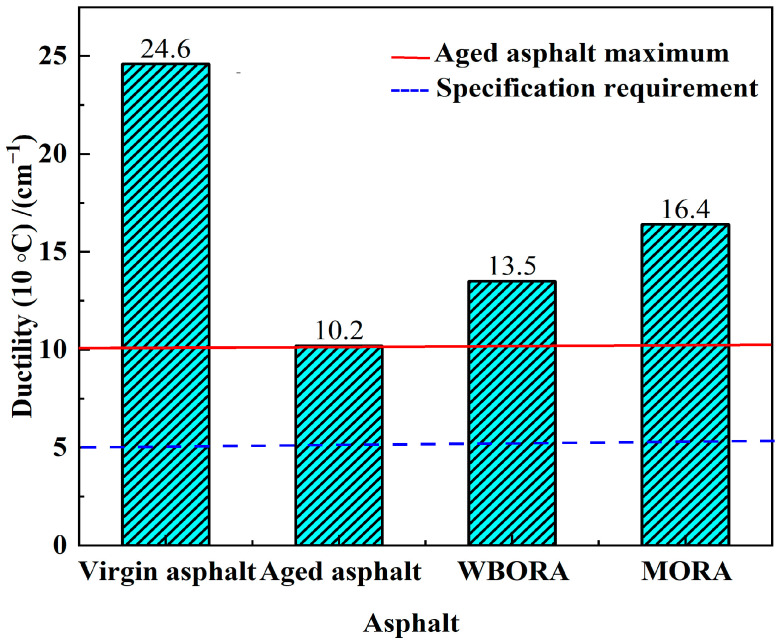
Ductility point of virgin asphalt, aged asphalt, WBORA, and MORA binders. The solid red line indicates the maximum ductility of aged asphalt (10.2 cm), while the blue dashed line represents the minimum specification requirement (5 cm) according to JTG F40-2004.

The ductility properties, serving as a critical indicator of asphalt’s deformation resistance [27], demonstrate significant variations among the tested samples. Figure 6 demonstrates that both regenerated asphalt specimens (WBO: 16.4 cm; MO: 13.5 cm) exhibited significantly greater ductility than aged asphalt (10.2 cm) at 10 °C (*p* < 0.05), confirming the rejuvenator’s effectiveness in restoring low-temperature performance. Notably, ductility increments diverged significantly (3.3 cm for WBORA vs. 6.2 cm for MORA), attributable to distinct regeneration mechanisms: WBORA primarily functions through chemical rejuvenation, where polar bio-oil constituents form hydrogen bonds and dipole interactions with oxygen-containing groups (C=O, S=O) in aged asphalt [28], selectively replenishing lost medium-molecular-weight components and partially reconstructing fractured molecular networks; conversely, MORA operates via physical rejuvenation as low-molecular-weight MO saturates penetrate asphaltene aggregates, weakening intermolecular forces to reduce viscosity and enhance macromolecular chain mobility. Critically, neither approach fully restored virgin asphalt ductility—WBORA’s chemical repair cannot cleave formed C-C/C-S cross-links [29], while MORA merely reduces viscosity without reconstructing the native colloidal structure.

### 3.2. Rotational Viscosity

**Figure 7:**
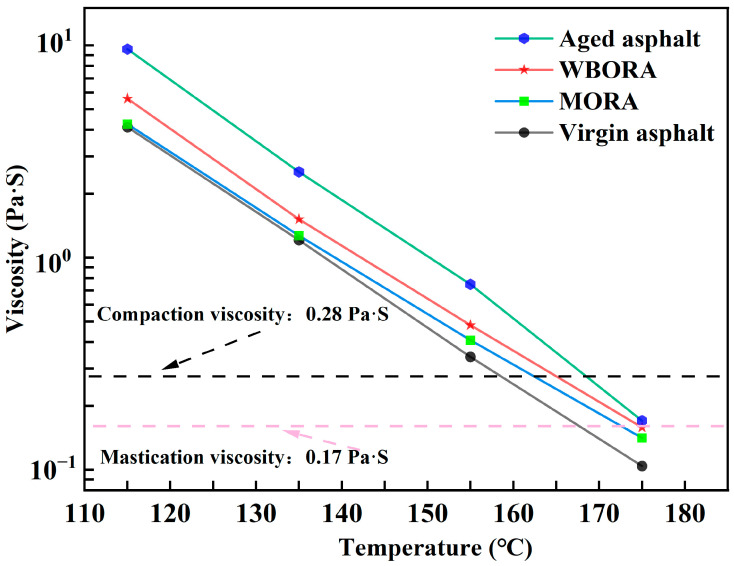
Viscosity of virgin asphalt, aged asphalt, WBORA, and MORA binders.

As evidenced in Figure 7, both recycled asphalts exhibit reduced viscosity compared with aged asphalt. This reduction occurs because aging [30] promotes oxidative polymerization—increasing molecular weight and asphaltene aggregation—which elevates viscosity significantly. Rejuvenators disrupt this rigid oxidized network through distinct mechanisms: WBO achieves controlled viscosity reduction via chemical repair, whereas mineral oil enables substantial viscosity decline through physical dilution. Notably, MORA approaches virgin asphalt viscosity levels more closely than WBORA. This optimal viscosity restoration significantly improves workability during construction operations by lowering mixing and compaction temperatures. Consequently, while both rejuvenation strategies effectively counter aging-induced viscosity elevation, MORA’s physical mechanism offers superior practical applicability for asphalt recycling.

### 3.3. Fourier Transform Infrared (FT-IR)

**Figure 8:**
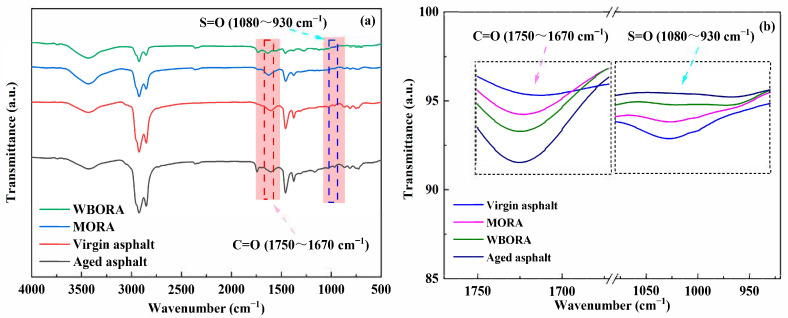
Infrared spectra (FT-IR) of different binders. (**a**) Virgin asphalt, aged asphalt, WBORA, and MORA binders; (**b**) Effect of rejuvenators on the evolution of characteristic functional groups in aged asphalt.

To further elucidate rejuvenation mechanisms, Fourier transform infrared (FT-IR) spectroscopy was used to analyze the chemical composition of virgin, aged, and regenerated asphalts, as presented in Figure 8.

FT-IR analysis of characteristic peaks (C=O at 1740 cm^−1^, S=O at 1030 cm^−1^) [31] reveals microstructural evolution during asphalt aging and regeneration. As shown in Figure 8a, intensified carbonyl peaks in aged asphalt confirm oxidative degradation, while subsequent peak attenuation upon rejuvenator addition indicates effective mitigation of aging effects. Notably, Figure 8b demonstrates significantly greater carbonyl reduction in WBORA compared with MORA, which is attributed to WBORA’s dual mechanisms: antioxidant-enabled oxidation inhibition and esterification between its carboxylic acids and carbonyl groups—distinct from the physical action of mineral oil. However, residual peak intensities indicate that neither approach fully restores virgin binder characteristics, suggesting rejuvenation primarily achieves rebalancing of physical components [32] rather than complete chemical reversal of aging damage.

It should be noted that for the FT-IR measurements, the transmittance mode with the KBr pellet technique is not ideal for direct comparison of band intensities, as unavoidable slight weight variations and carbon content (which absorbs transmitted beams) can lead to differences in overall spectral intensities. Instead, ATR mode is more appropriate for such measurements; alternatively, normalizing spectra to a stably expected band is recommended for improved comparability.

### 3.4. Thermogravimetric Test (TG)

**Figure 9:**
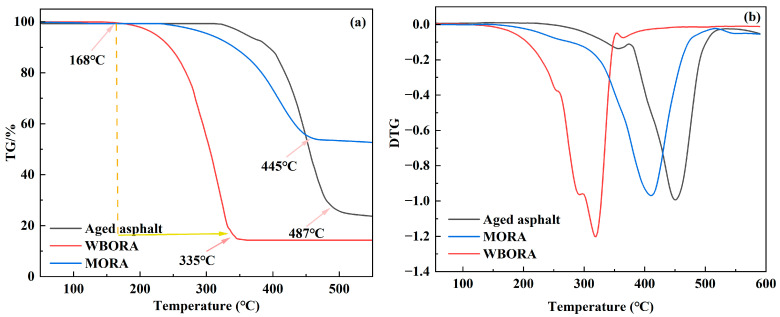
TG and DTG curves of aged asphalt, BORA, and MORA binders: (**a**) TG curves; (**b**) DTG curves.

Asphalt is subjected to elevated temperatures during the mixing process, making the assessment of recycled asphalt’s rheological high-temperature stability critically important [33]. Thermogravimetric analysis (TGA) was employed to evaluate the high-temperature stability of aged and recycled asphalts, as presented in Figure 9.

Figure 9a,b demonstrate a two-stage thermal decomposition process in aged and recycled asphalts [34], with distinct temperature boundaries and curve features that define each stage. The first stage corresponds to the initial gradual mass loss: for recycled asphalts, this stage extends up to ~300 °C, evident in TG curves as a slow, steady mass loss and in DTG curves (Figure 9b) as a distinct, albeit moderate peak within 200–300 °C (most prominent in WBORA), directly attributable to the gradual release of volatile rejuvenator components. For aged asphalt, the initial gradual mass loss persists up to ~400 °C, reflected in TG curves by low-rate mass loss over this range and in DTG curves by the absence of distinct peaks below 300 °C (indicating depletion of light fractions) and only weak, broad signals between 300 and 400 °C (consistent with the slow breakdown of stable heavy fractions). The second stage involves accelerated decomposition: for recycled asphalts, this begins beyond ~300 °C, marked by a sharp increase in mass loss rate on TG curves and a left-shifted main peak in DTG curves (relative to aged asphalt); for aged asphalt, accelerated decomposition starts beyond ~400 °C, characterized by a broadened DTG peak spanning 400–500 °C, corresponding to the rapid decomposition of polymerized heavy fractions. Both stages conclude with stabilization following the completion of their respective decomposition processes.

### 3.5. Differential Scanning Calorimetry (DSC)

**Figure 10:**
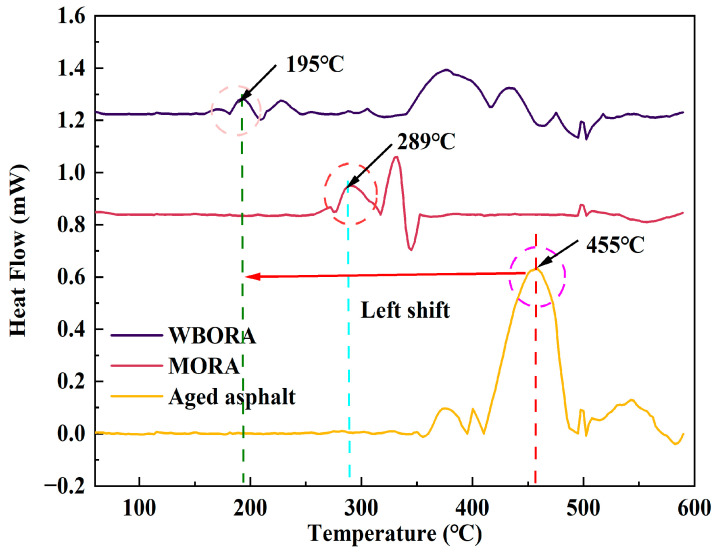
DSC curves of aged asphalt, WBORA, and MORA.

As evidenced in Figure 10, WBORA exhibits reduced peak intensity with oxidation onset temperatures at 190–210 °C, attributable to the partial restoration of free volume by polar molecules. Conversely, MORA displays onset peaks at 280–300 °C, where low-molecular-weight hydrocarbons enhance macromolecular chain mobility. This divergence in oxidation initiation temperatures stems from fundamentally distinct mechanisms: WBORA’s hydrogen bonding between polar constituents and asphaltenes versus MORA’s crystallization/melting transitions of saturated alkanes. These differential molecular interactions directly underlie the characteristic peak shifts and ultimately explain macroscopic performance variations in recycled asphalts.

It should be noted that the DSC measurements may have limitations. The observed noise in the thermograms could be related to the crucible setup used. According to literature, DSC measurements for asphalt and bitumen typically require crucibles with pierced lids to allow volatile components to escape, which helps mitigate such noise; however, this setup was not employed in the present study. Alternatively, stainless steel double-sealed crucibles can be used, though they are relatively expensive. These methodological considerations may affect the precision of the observed peak characteristics.

### 3.6. Rheological Properties (DSR)

#### 3.6.1. Temperature Sweep

**Figure 11:**
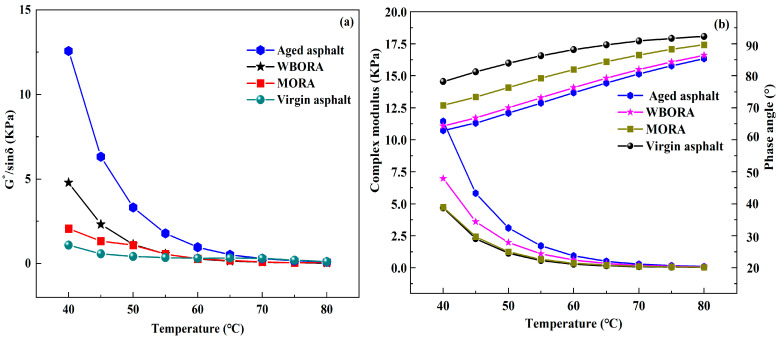
Results of temperature scanning tests: (**a**) The rutting factor of virgin, aged asphalt, WBORA, and MORA at different temperatures; (**b**) The complex modulus of virgin, aged asphalt, WBORA, and MORA at different temperatures.

Dynamic shear rheometry (DSR) was employed to characterize the rheological properties of virgin, aged, and recycled asphalts. Elevated rutting factor (G*/sinδ) values—corresponding to increased complex modulus (G*) and reduced phase angle (δ)—indicate enhanced elastic recovery performance [35]. Figure 11 comparatively presents the rutting factors and complex moduli of these three asphalt states.

Figure 11a demonstrates that asphalt aging elevates the rutting factor (G*/sinδ) due to asphaltene polymerization, molecular weight increase, and enhanced elastic dominance, while rejuvenator incorporation reduces G*/sinδ—indicating restored ductility—with WBORA exhibiting smaller reductions than MORA due to superior retention of elastic components. Correspondingly, Figure 11b reveals aging-induced complex modulus (G*) elevation from molecular growth and restricted mobility, improving deformation resistance; rejuvenators significantly reduce G* yet maintain values above virgin asphalt, enhancing high-temperature stability, where MRA displays greater G* than WBORA at identical temperatures owing to fundamentally distinct mechanisms: chemical elasticity repair WBORA versus physical modulus reduction MORA. Concurrently, aging reduces phase angle (δ), signifying embrittlement, while rejuvenators increase δ to recover viscoelastic balance—though all recycled asphalts exhibit lower δ than virgin binder, their divergent high-temperature performance (consistent with G* and rutting factor trends) directly correlates with rejuvenator-specific chemorheological reconstruction.

#### 3.6.2. Frequency Sweep

**Figure 12:**
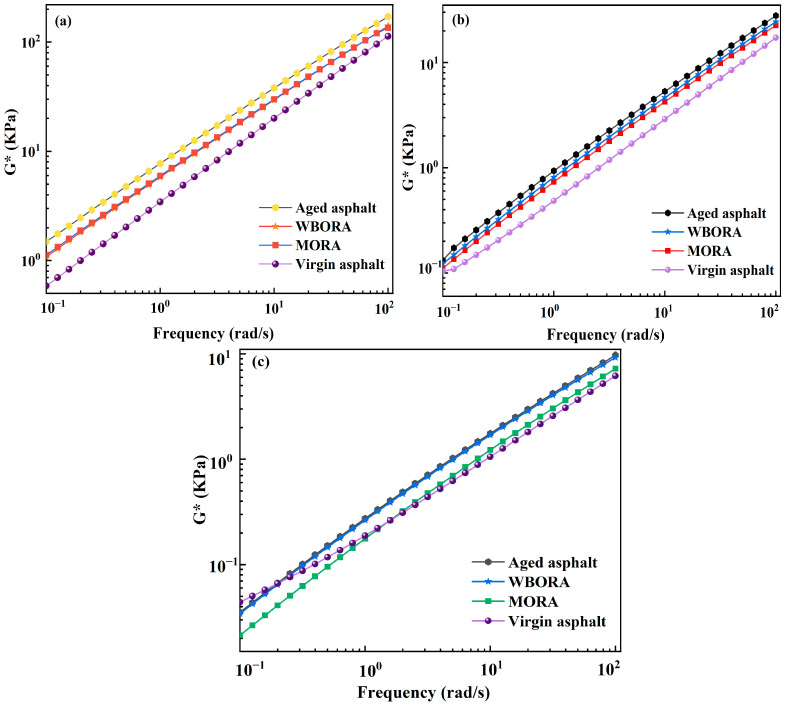
Results of frequency scanning tests: (**a**) The rutting factor of virgin, aged asphalt, WBORA, and MORA at 58 °C; (**b**,**c**) the complex modulus of virgin, aged asphalt, WBORA, and MORA at 64 °C and 70 °C.

Dynamic frequency sweeps via DSR simulate asphalt’s rheological response to traffic speeds through oscillatory loading at varying frequencies [36], as presented in Figure 12.

Figure 12 demonstrates that complex modulus (G*) universally increases with frequency, while rejuvenator incorporation reduces G*—confirming asphalt softening consistent with softening point trends. Notably, temperature-dependent master curve shifts toward lower frequencies are more pronounced in MORA. This divergence stems from fundamentally distinct mechanisms: WBORA achieves partial elastic network reconstruction through polar constituents, restoring G* while preserving phase angle equilibrium; conversely, MORA undergoes preferential viscosity reduction via physical dilution, accelerating G* decline at elevated temperatures. Cole–Cole plot deconvolution further evidences WBORA’s restored viscoelastic balance versus MORA asphalt’s viscosity-dominated behavior.

## 4. Conclusions

This study synthesizes a low-viscosity rejuvenator from restaurant waste vegetable oil via transesterification technology. Two resultant rejuvenators (WBORA and MORA) were homogenized with aged asphalt to produce recycled binders. Employing a penetration-based optimization approach, the optimal dosage was determined when aged asphalt penetration restored to virgin binder values (0.1 mm). At this dosage, systematic evaluations were conducted on the following:

(1) Conventional performance tests confirm that both rejuvenated asphalts exhibit softening effects. Viscosity of aged asphalt decreases progressively with rejuvenator incorporation, ensuring adequate workability during construction operations.
(2) FT-IR spectroscopy reveals increased carbonyl (C=O) and sulfoxide (S=O) indices in aged asphalt versus virgin binder. These indices attenuate upon rejuvenator addition, with WBORA demonstrating superior carbonyl peak reduction. MORA shows no chemical interactions—only physical blending with aged asphalt constituents.
(3) Thermogravimetric analysis indicates rejuvenators compromise high-temperature stability but enhance low-temperature performance. The left-shifted oxidation onset peaks (WBORA: 190–210 °C; MORA: 280–300 °C) evidence WBORA asphalt’s superior rheological restorative efficacy, concurrently preserving high-temperature stability and low-temperature ductility.
(4) Combined temperature-frequency sweeps show neither rejuvenated asphalt nor fully restored virgin asphalt’s rheological complex modulus (G*) or phase angle (δ). This stems from fundamentally distinct regeneration mechanisms: MORA primarily achieves physical restoration, whereas WBORA facilitates chemical reconstruction—as validated via Cole–Cole plot divergence.

## Figures and Tables

**Table 1:** Conventional properties of virgin asphalt.

Test	Result	Specification
Penetration (25 °C)/(0.1 mm)	65	60–80
Softening point (≥)/°C	49	45
Ductility (10 °C)/cm	25	≥20

**Table 2:** Physicochemical properties of WBO and MO.

Indexes	Viscosity (60 °C, mPa·s)	Density (g/cm^3^)	Flash Point (°C)	Mass Fraction of Saturates /%	Mass Fraction of Aromatics/%
WBO	60	0.893	209	4.32	85.51
MO	57	0.876	318	4.91	83.32
Specification	50–500	0.8–1.05	≥200	≤30	Measured

**Table 3:** Conventional properties of aged asphalt.

Test	Result	Specification
Penetration (25 °C)/(0.1 mm)	29	60–80
Softening point (≥)/°C	81	≥45
Ductility(10 °C)/cm	10	≥20

## Data Availability

The original contributions presented in this study are included in the article. Further inquiries can be directed to the corresponding authors.
